# Expression of Human Epidermal Growth Factor Receptor-2 Status and Programmed Cell Death Protein-1 Ligand Is Associated With Prognosis in Gastric Cancer

**DOI:** 10.3389/fonc.2020.580045

**Published:** 2021-02-01

**Authors:** Huifang Lv, Junling Zhang, Keran Sun, Caiyun Nie, Beibei Chen, Jianzheng Wang, Weifeng Xu, Saiqi Wang, Yingjun Liu, Xiaobing Chen

**Affiliations:** ^1^ Department of Oncology, The Affiliated Cancer Hospital of Zhengzhou University, Henan Cancer Hospital, Zhengzhou, China; ^2^ Medical Department, 3D Medicines Inc., Shanghai, China; ^3^ Department of Surgery, The Affiliated Cancer Hospital of Zhengzhou University, Henan Cancer Hospital, Zhengzhou, China

**Keywords:** HER-2, PD-L1, prognosis, gastric cancer, CD8+T cells

## Abstract

**Background:**

PD-L1 and HER-2 are routine biomarkers for gastric cancer (GC). However, little research has been done to investigate the correlation among PD-L1, HER-2, immune microenvironment, and clinical features in GC.

**Methods:**

Between January 2013 and May 2020, a total of 120 GC patients treated with chemotherapy were admitted to Henan Tumor Hospital. We retrospectively identified PD-L1, HER-2 level before chemotherapy and abstracted clinicopathologic features and treatment outcomes. Univariate and multivariate survival analyses were performed to assess the relationship between PD-L1/HER-2 levels and progression-free survival (PFS). The mRNA and tumor microenvironment of 343 patients with GC from The Cancer Genome Atlas (TCGA) were used to explore the underlying mechanism.

**Results:**

We retrospectively analyzed 120 patients with gastric cancer, including 17 patients with HER-2 positive and 103 patients with HER-2 negative GC. The results showed that the expression of PD-L1 was closely correlated with HER-2 (P = 0.015). Patients with PD-L1/HER-2 positive obtained lower PFS compared to PD-L1/HER-2 negative (mPFS: 6.4 *vs*. 11.1 months, P = 0.014, mPFS: 5.3 *vs*. 11.1 months, P = 0.002, respectively), and the PD-L1 negative and HER-2 negative had the best PFS than other groups (P = 0.0008). In a multivariate model, PD-L1 status, HER-2 status, tumor location, and tumor differentiation remained independent prognostic indicators for PFS (P < 0.05). The results of database further analysis showed that the proportion of PD-L1+/CD8A+ in HER-2 negative patients was higher than that in HER-2 positive patients (37.6 *vs* 20.3%), while PD-L1−/CD8A− was significantly higher in HER-2 positive patients than HER-2 negative patients (57.8 *vs*. 28.8%). In addition, it showed that not only CD4+T cells, macrophages, and CD8+T cells, but also the associated inflammatory pathways such as IFN-*γ*/STAT1 were associated with HER-2.

**Conclusion:**

HER-2 status could predict the efficacy of immune checkpoint inhibitors, and HER-2 status combined with PD-L1 level could predict the prognosis of GC patients.

## Introduction

Gastric cancer (GC) is a common malignant tumor in the digestive tract, ranking the second in the global mortality rate of malignant tumors, and more than 50% of new cases are from developing countries (1). The 5-year overall survival rate of metastatic GC is only 5–20% (2). Human epidermal growth factor receptor-2 (*HER-2*, also known as ERBB2) is a transmembrane receptor tyrosine kinase, and *HER-2* expression is significantly up-regulated in approximately 6–23% GC tissues (3–5). Since trastuzumab combined with chemotherapy became the standard treatment for advanced GC with positive HER-2 (ToGA study), a significant increase was needed for HER-2 assessment for GC (6). In breast cancer, HER-2 amplification and overexpression are associated with low prognosis, high mortality, and high recurrence and metastasis (7–9). However, the prognostic value of HER-2 in GC remains controversial. Some studies have shown that HER-2 positive patients have a high survival rate (10–12). In addition, HER-2 positive patients are correlated with serous membrane infiltration, lymph node metastasis, disease stage, distant metastasis, and other clinicopathological characteristics (13, 14). Other studies have shown no correlation between *HER-2* expression and survival (15–17).

The interaction of programmed cell death protein-1 (PD-1) and its ligand (PD-L1) with immune cells and tumor cells limits the T-cell-mediated immune response (18). Immune checkpoint blocking of anti-PD-1 or anti-PD-L1 antibodies is the latest treatment for a variety of cancers, including non-small cell lung cancer (19–21), melanoma (22), bladder cancer (23), and kidney cancer (24). In early clinical studies, PD-1 inhibitors in the treatment of metastatic gastric cancer, such as pembrolizumab (25) and nivolumab (26), have been reported to have good efficacy. Current studies have shown that the expression level of PD-L1 in tumor tissue could be used to predict the efficacy of anti-PD-1 treatment (27); not only in patients with high expression of PD-L1 will it be effective, but also in patients with low expression of PD-L1. Therefore, it is essential to find the best biomarkers for GC in order to provide predictive information about the treatment response and ultimately improve the treatment outcome. The expression level of PD-L1 and the status of HER-2 are two important pathological characteristics of gastric cancer patients. Although some studies focused on the expression of PD-L1 and HER-2 in gastric cancer, the results of these studies are not consistent. Some researchers have found that expression of PD-L1, a potential biomarker for the immunotherapy response, was observed in HER-2 positive and negative patients to a similar extent, and its presence was not influenced by the HER-2 status (28). However, it has also been studied that the PD-L1 expression in GC is significantly correlated with HER2-negative status (29). Therefore, the relationship between HER-2 and PD-L1 state and what role the immune microenvironment plays in the prognosis of GC patients are still not clear.

In order to demonstrate the association between HER-2 and PD-L1 status, we analyzed the data from the largest available cohort of GC with both clinical and survival data. The immune microenvironment and PD-L1 mRNA from The Cancer Genome Atlas were also analyzed to explore the possible underlying mechanism.

## Materials and Methods

### Study Design and Clinical Data Collection

We retrospectively reviewed 120 GC patients at the Affiliated Cancer Hospital of Zhengzhou University between January 2013 and May 2020. All patients were confirmed by two pathologists and the histological diagnoses were without discrepancy. Patients without any signs of distant metastasis preferably received neoadjuvant treatment, which was followed by surgical resection of the tumor. After an adjuvant chemotherapy period, routine control visits with computed tomography (CT) scans were performed. Patients with typical signs of distant metastasis underwent palliative chemotherapy. Biopsy or resection samples were used to detect PD-L1 and HER-2 expression. If the tumor was HER-2 positive, trastuzumab was added to the treatment schedule. Trastuzumab was administered by intravenous infusion at a dose of 8 mg/kg on day 1 of the first cycle, followed by 6 mg/kg every 3 weeks until progression of the disease, the occurrence of unacceptable toxicity, or the patient’s refusal. After administration of two cycles of chemotherapy or trastuzumab containing treatment, the size of the tumor was investigated by CT imaging and assessed using the Response Evaluation Criteria in Solid Tumors version 1.1 (RECIST 1.1). The following clinical characteristics were abstracted: age, sex, HER-2 status, PD-L1 status, tumor differentiation degree, lauren classification, treatment. The follow-up information was conducted *via* medical records plus telephone interview, and the following information was obtained: disease-free survival (DFS) and progression free survival (PFS).

In addition, the PD-L1 mRNA data and immune microenvironment of 343 patients with gastric cancer (GC) were sourced from The Cancer Genome Atlas (TCGA) (www.cbioportal.org).

The study was approved by relevant regulatory and independent ethics committee of the Henan Tumor Hospital and done in accordance with the Declaration of Helsinki and the International Conference on Harmonization Good Clinical Practice guidelines.

### Immunohistochemical Staining and Evaluation

Representative sections of each surgical tumor resection or biopsy specimens were stained with PD-L1 antibody (SP263, Ventana) and VENTANA HER-2/neu rabbit monoclonal antibody (Clone 4B5, Ventana). Omission of primary antibody and substitution by non-specific immunoglobins were used as negative controls. The immunoreactivity of PD-L1 was evaluated according to combined positive score (CPS). CPS was calculated by dividing the number of PD-L1 positive tumor cells, lymphocytes and histiocytes by the total number of vital tumor cells and then multiplying the result by 100. Specimens in which PD-L1 staining was observed in CPS >1 were considered PD-L1 positive. And CPS ≤1 was regarded as PD-L1 negative. IHC 3+ or IHC 2+ was defined as HER-2 positive.

### Fluorescence In Situ Hybridization

**Figure 1:**
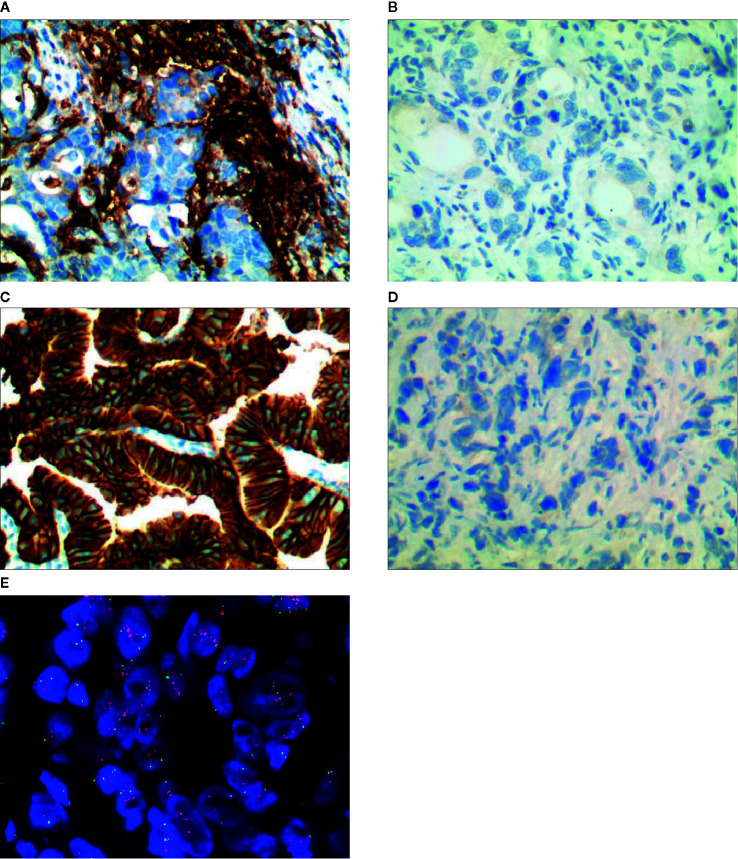
Representative images of PD-L1 and HER-2 immunostaining/FISH results, **(A)** PD-L1 positive, **(B)** PD-L1 negative, **(C)** HER-2 positive by immunostaining, **(D)** HER-2 negative by immunostaining, **(E)** HER-2 positive by FISH.

When the result of IHC was 2+/3+, the amplification level of HER-2 was detected. PathVysion DNA Probe kit was used for the analysis of FISH according to the manufacturer’s protocol. The positive results from FISH were defined as a HER-2: CEP17 ratio ≥2.0. Examples of HER2 FISH positive and negative were shown in **Figures 1E, F**. According to the standards of the European Medicines Agency, HER-2 positive was defined as any case of IHC 3+ or IHC 2+ with a positive FISH result, while any case of IHC 0, IHC 1+ or IHC 2+ with a negative FISH result is considered HER-2 negative.

### Statistical Analyses

Progression free survival (PFS) was defined as the time from the date of first line therapy administration to the progression of cancer, or death from any cause. PFS was calculated using the Kaplan–Meier method. Correlation analyses were performed using the two-sided chi-squared test or the Fisher exact test. Variables with significant *P* values or interest were included into multivariate logistic regression. For all analyses, *P* value <0.05 was considered to be statistically significant, and a confidence interval of 95% was used (95% CI). All statistical analyses were performed using SPSS22.0 software (SPSS, Inc., Chicago, IL, USA).

## Results

### Patient Baseline Clinical Features

We retrospectively analyzed 120 patients with gastric cancer in our hospital, including 17 patients with HER-2 positive and 103 patients with HER-2 negative GC (**Table 1**). There were 32 patients with PD-L1 positive and 88 patients with PD-L1 negative (**Figure 1**). 57.5% were male and 42.5% GC patients were ≥60 years. The results showed that the expression of PD-L1 was closely correlated with HER-2 status, with statistical significance (*P* = 0.015, as shown in **Table 1**).

**Table 1:** Clinicopathologic and baseline clinical features of gastric cancer patients.

Characteristics	PD-L1 Positive (n = 32)	PD-L1 Negative (n =88)	*P*
**Sex**			
Male	21	48	0.3039
Female	11	40	
**Age**			
≥60	14	37	>0.9999
<60	18	51	
**Histological differentiation**			
Moderate	7	17	0.7031
Poor	19	48	
NOS	6	23	
**Lauren Classification**			
Diffuse	4	18	0.1809
Intestinal	2	8	
Mixed	5	4	
Unknown	21	58	
**Tumor Location**			
Body	12	31	0.2573
Antrum	5	11	
Cardia, gastric fundus	15	36	
Unknown	0	10	
**T stage (%)**			
T1	0	4	0.336
T2	4	7	
T3	7	30	
T4	6	9	
Tx	15	38	
**N stage (%)**			
N0	4	13	0.8118
N1	2	6	
N2	2	12	
N3	8	18	
Nx	16	39	
**M stage (%)**			
M0	15	54	0.1476
M1	17	31	
Mx	0	3	
**HER-2 status**			
Positive.	9	8	0.0153
Negative	23	80	

### Association Between Programmed Cell Death Protein-1 Ligand/Human Epidermal Growth Factor Receptor-2 Status and Survival Outcomes

**Figure 2:**
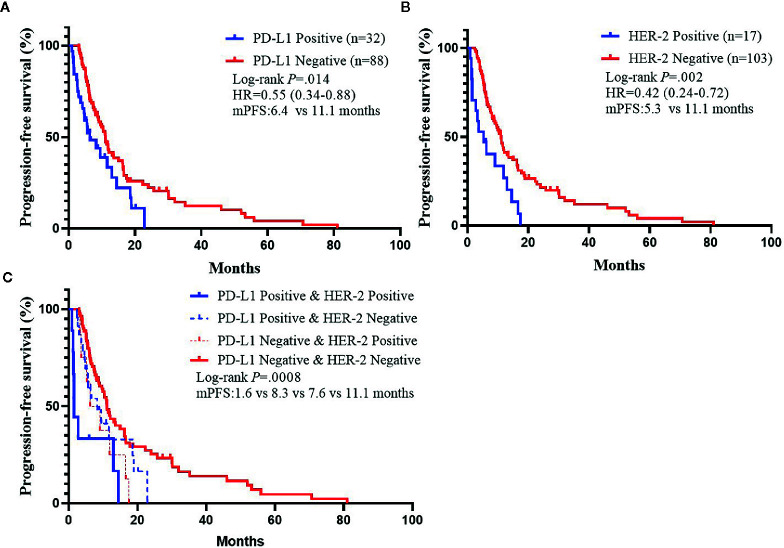
Kaplan–Meier Estimates of Progression-Free Survival by PD-L1 Status or *HER-2* status. **(A)** Kaplan–Meier survival curves of PFS by PD-L1 status. **(B)** Kaplan–Meier survival curves of PFS by *HER-2* status. **(C)** Kaplan–Meier survival curves of PFS by PD-L1 and HER-2 status.

We analyzed whether PD-L1/HER-2 status was associated with the survival outcomes of chemotherapy in advanced GC. Patients with PD-L1 positive obtained lower PFS compared to PD-L1 negative (mPFS: 6.4 *vs.* 11.1 months, *P* = 0.014, **Figure 2A**). The similar results were in HER-2 negative (mPFS: 5.3 *vs.* 11.1 months, *P* = 0.002, **Figure 2B**). And the PD-L1 negative and HER-2 negative had the best PFS than the other groups (*P* = 0.0008, **Figure 2C**). In the present study, univariable analysis revealed significant association between poorer PFS and PD-L1 status, HER-2 status, tumor location in body, while there was no relation between PFS and age, sex, lauren classification and tumor differentiation (**Table 2**). In a multivariate model, PD-L1 status, HER-2 status, tumor location, and tumor differentiation remained independent prognostic indicators for PFS (**Table 1**, *P* < 0.05).

**Table 2:** Univariate and multivariate analyses of progression-free survival.

Parameter	Univariate analysis			Multivariate analysis		
	HR	95% CI	*P*	HR	95% CI	*P*
Sex Male *vs* Female	0.769	0.509–1.162	0.213			
Age ≥ 60 *vs <*60	0.825	0.540–1.260	0.373			
Tumor differentiation Moderate *vs*. poorly	0.629	0.375–1.056	0.080	0.444	0.25–0.777	0.004
LAUREN Diffuse *vs* intestinal	0.928	0.522–1.649	0.799	2.009	1.257–3.210	0.004
Tumor location Body *vs* antrum	1.614	1.034–2.519	0.035			
PD-L1 status Negative *vs* Positive	0.547	0.339–0.883	0.014	0.596	0.364–0.975	0.039
HER-2 status Negative *vs* Positive	0.416	0.240–0.722	0.002	0.280	0.149–0.525	0.000

### Association Between Human Epidermal Growth Factor Receptor-2 Status and Programmed Cell Death Protein-1 Ligand mRNA Expression

**Figure 3:**
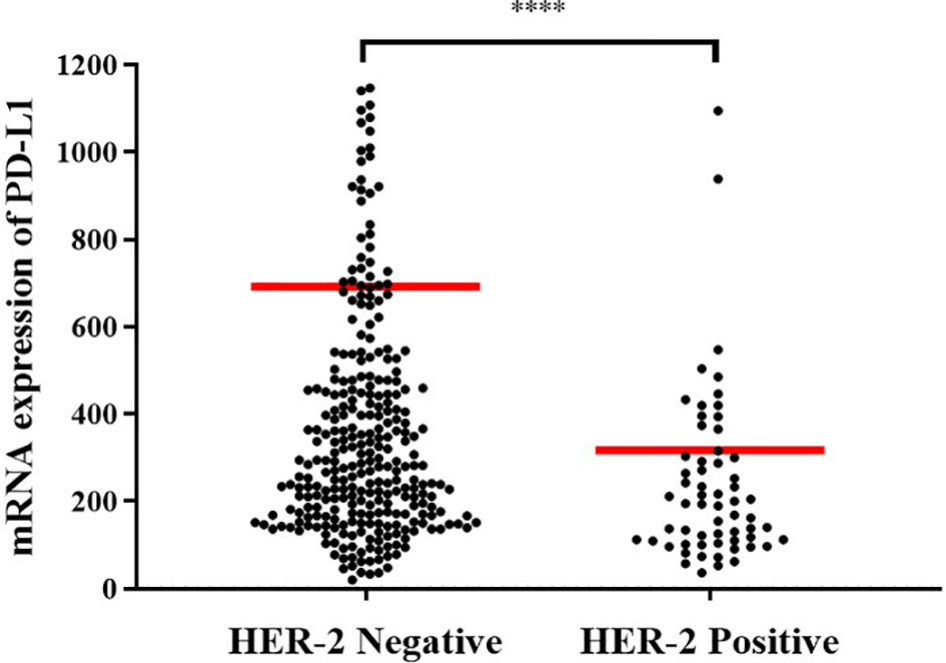
Expression levels of PD-L1 mRNA in different *HER-2* gene states. *HER-2* amp represents *HER-2* amplification; *HER-2* non-amp indicates *HER-2* non-amplification. ****P < 0.0001.

In order to explore the mechanism of potential, we first analyzed whether HER-2 status was associated with the PD-L1 mRNA expression in GC. It showed that the expression of PD-L1 was higher in HER-2 negative GC, but decreased in HER-2 positive GC (**Figure 3**, *P* < 0.0001).

### Association Between Programmed Cell Death Protein-1 Ligand Status and Tumor-Infiltrating Lymphocyte

**Figure 4:**
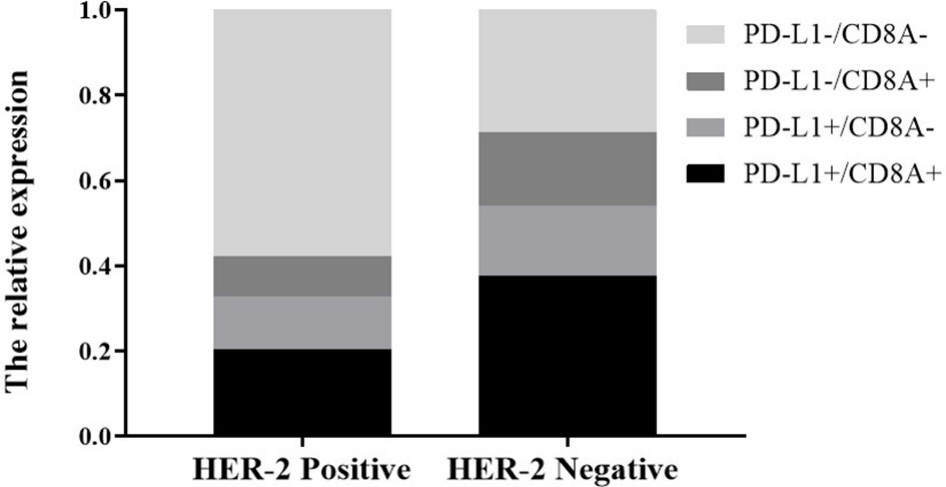
Effect of *HER-2* status on tumor immune typing.

tAccording to the classification of PD-L1 and TIL, tumors were divided into PD-L1^−^/TIL^−^, PD-L1^+^/TIL^+^, PD-L1^+^/TIL^−^ and PD-L1^−^/TIL^+^, among which PD-L1^+^/TIL^+^ was considered to be the most suitable state for immunotherapy (27). We further analyzed the effect of *HER-2* on the distribution of PD-L1/CD8A in TCGA data. In *HER-2* amplified patients, the proportions of PD-L1^+^/CD8A^+^, PD-L1^+^/CD8A^−^, PD-L1^−^/CD8A^+^ and PD-L1^−^/CD8A^−^ were 20.3, 12.5, 15.6, and 57.8%, respectively. The proportions of PD-L1^+^/CD8A^+^, PD-L1^+^/CD8A^−^, PD-L1^−^/CD8A^+^, and PD-L1^−^/CD8A^−^ in patients without HER-2 amplification were 37.6, 16.4, 17.2, and 28.8%, respectively (see **Figure 4**, *P* < 0.001). The results indicated that the ratio of PD-L1^+^/CD8A^+^ was significantly increased in patients without HER-2 amplification, while the ratio of PD-L1^−^/CD8A^−^ was the highest in patients with HER-2 amplification. This result further suggests that immunotherapy may be more effective for patients with HER2-negative GC, while patients with HER2-positive GC have a poorer prognosis, and combination therapy may be effective.

### Association Between Human Epidermal Growth Factor Receptor-2 Status and Immune Cell Infiltration

**Figure 5:**
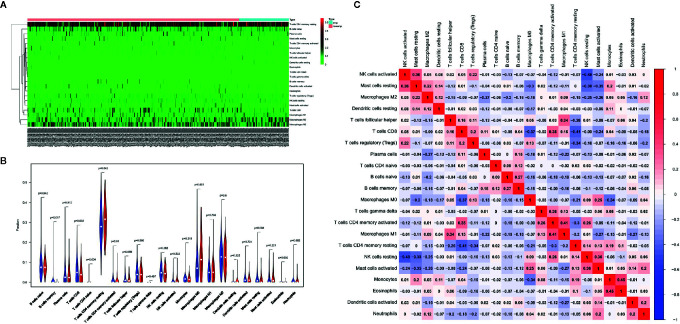
Effects of HER-2 status and immune cell infiltration. **(A)** The heat map of immune cells in GC. **(B)** The proportion of 22 immune cells in GC. **(C)** The correlation between the immune cells infiltration in GC by Pearson analyzed.

In the following experiments, we attempted to explore the effect of HER-2 status on immune cell infiltration. Through deconvolution of 574 labeled gene expression values, the proportions of 22 kinds of immune cells in GC tissues in TCGA database were analyzed by CIBERSORT. The results showed that the proportion of resting state memory CD4+ T cells was the highest in GC samples, followed by macrophages. CD8+ T cells and memory B cells were highly expressed in the non-amplified HER-2 group, while resting state memory CD4+ T cells and M0 macrophages were highly expressed in the amplified HER-2 group (**Figures 5A, B**).

Pearson correlation analysis showed that there was no significant correlation among immune cells infiltration (**Figure 5B**). M1-type macrophages were moderately correlated with activated memory CD4+ T cells (r = 0.41), while resting memory CD4 + T cells were negatively correlated with CD8 + T cells (r = −0.41). The results showed that the proportion of resting CD4+ T cells in the immune microenvironment of HER-2 amplified patients was high, and the proportion of activated memory CD4+ T cells was low, suggesting that CD4+ T cells in the immune microenvironment were not activated, which reduced the flooding effect of CD8+ T cells in the immune microenvironment, leading to decreased infiltration of CD8+ T cells.

### Association Between Human Epidermal Growth Factor Receptor-2 Status and Cytokines

**Figure 6:**
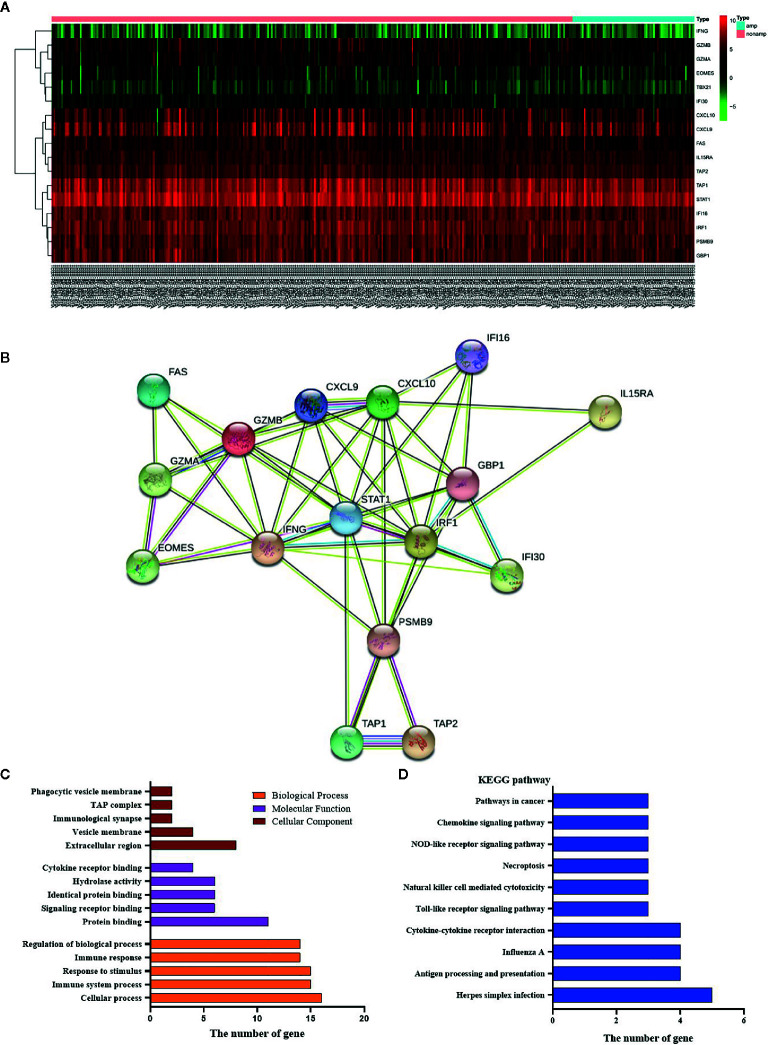
Effects of *HER-2* status and cytokines. **(A)** The heat map of cytokines in GC. **(B)** Interaction mapping of different gene in *HER-2* Amp and non-amp group. **(C)** Gene distribution based on GO analysis. **(D)** KEGG pathway analysis of differential genes.

By comparing HER-2 amplification and non-amplification groups, significant changes in some cytokines were found as shown in **Figure 6A**, and INF-*γ* was significantly decreased. By using an online system (https://string-db.org/cgi/network.pl?taskId=lP6ij62YlPsZ), we found that the *STAT1* had a close reciprocal relationship with *IFN-γ*. Biological process analysis of cytokines showed that they are mainly involved in immune responsibility-related reactions (**Figure 6B**). At the same time, KEGG pathway analysis was also carried out, and it was found that antigen processing and presentation, natural killer cell mediated cytotoxicity and Toll-like receptor signaling pathway were included (**Figure 6D**). Therefore, we could find that HER-2 status is closely related to the immune response. Amplification of HER-2 may negatively regulate the immune response of GC and further affect the anti-tumor effect, which explains why immunotherapy for HER-2 positive GC patients is not effective.

## Discussion

As immunotherapy has ushered in a new era in the treatment of GC, PD-1 inhibitors have become the standard treatment for PD-L1 positive advanced GC, and further studies on immune-related biomarkers and their interactions with other cancer-related pathways are necessary. In our study, we investigated the potential correlation between HER-2 and PD-L1 expression and their relationship with clinical characteristics and prognosis in patients with GC.

Immunotherapy, especially immune checkpoint blockade, has become a promising cancer treatment. Immune checkpoint inhibitors, such as anti-PD-1 and anti-PD-L1, have been approved by the Food and Drug Administration (FDA) for the treatment of various types of cancer, resulting in durable tumor regression and prolonged survival (30, 31). It has also been shown that blocking PD-L1 could improve the immune function of tumor-specific effector T cells when interacting with target tumor cells *in vitro* (32). However, the relationship between PD-L1 expression and prognosis in GC is still controversial. Some studies found that the prognosis of GC patients with PD-L1 positive was significantly improved (33). On the contrary, other researchers have shown that high PD-L1 expression was a significant poor prognostic factor (34). In this study, we found that positive PD-L1 in GC tissues was associated with poor prognosis of PFS. This finding is consistent with previous research results (35). A reasonable hypothesis for the poor clinical efficacy of PD-L1 positive tumors is that the up-regulation of PD-L1 in immune cells inactivates cytotoxic T lymphocytes (CTLs), leading to host immune evasion.

Interestingly, we also found that the expression of PD-L1 was higher in HER-2 negative GC, but decreased in HER-2 positive GC which might lead to a novel treatment strategy. As in the ToGA study, only HER-2 positive patients can benefit from anti-HER-2 drug (5). Anti-PD-1/PD-L1 immunotherapy might become a potentially new treatment for HER-2 negative patients. Whether HER-2 could be used independently as an indicator to evaluate the disease progression and prognosis of GC patients was still a big controversy. A retrospective study found that HER-2 was highly expressed in GC and closely related to poor quality of life and short survival, indicating that HER-2 has a certain potential value in prognosis assessment of GC (12). Other research results showed that the high expression of HER-2 in GC tissues was only negatively correlated with the degree of tumor differentiation, while there was no difference in the distribution of other pathological characteristics related data such as gender, age, tumor size (36), which were similarly with our study.

More literature indicates that tumor microenvironment plays a critical role in cancer progression and treatment response (37). Not only compositions, but also the number of T cells, associated macrophages, and associated inflammatory pathways influenced the immune response and chemotherapy benefit at diagnosis (38–40). Based on the existence of tumor-infiltrating lymphocytes (TILs) and PD-L1 expression, we know that PD-L1+CD8+ was adaptive immune resistance. In our study, the ratio of PD-L1+/CD8A+, CD8+T cells, and B cells were highly expressed in the non-amplified HER-2 group and CD4+T cells and macrophages M0 were highly expressed in the amplified HER-2 group. In addition, immune responsibility-related reactions of biological process and a significant decrease in IFN-*γ* were found in HER-2 negative GC. Those also highlight the potential role of tumor microenvironment in GC and explain the fact that HER-2 negative patients are more suitable for immunotherapy.

Taken together, PD-L1 positive in tumor cells is correlated with worse prognosis in GC patients and is correlated positively with HER-2 positive. Our findings suggest that tumors expressing higher levels of PD-L1 are more aggressive and that administration of adjuvant chemotherapy should be considered for patients with these tumors.

## Data Availability Statement

The original contributions presented in the study are included in the article/supplementary material, further inquiries can be directed to the corresponding author.

## Ethics Statement

The study was approved by relevant regulatory and independent ethics committee of the Henan Tumor Hospital and done in accordance with the Declaration of Helsinki.

## Author Contributions

HL and XC designed the study. HL and JZ wrote the first draft of the manuscript. HL, KS, CN, BC, JW, WX, SW, and YL treated the patients and acquired data. HL and JZ analyzed the data. XC revised the manuscript. All authors contributed to the article and approved the submitted version.

## Funding

We would like to thank the financial support from the National Natural Science Foundation of China (No. 81472714), 1000 Talents Program of Central plains (No. 204200510023), Science and Technique Foundation of Henan Province (No. 202102310413), Medical Science and Technique Foundation of Henan Province (Nos. 2018020486 and SB201901101) and State Key Laboratory of Esophageal Cancer Prevention & Treatment (No. Z2020000X). 

## Conflict of Interest

JZ is an employee of Shanghai 3D Medicines Inc.

The remaining authors declare that the research was conducted in the absence of any commercial or financial relationships that could be construed as a potential conflict of interest.
