# Corrigendum: Expression of Human Epidermal Growth Factor Receptor-2 Status and Programmed Cell Death Protein-1 Ligand Is Associated With Prognosis in Gastric Cancer

**DOI:** 10.3389/fonc.2021.672599

**Published:** 2021-04-20

**Authors:** Huifang Lv, Junling Zhang, Keran Sun, Caiyun Nie, Beibei Chen, Jianzheng Wang, Weifeng Xu, Saiqi Wang, Yingjun Liu, Xiaobing Chen

**Affiliations:** ^1^ Department of Oncology, The Affiliated Cancer Hospital of Zhengzhou University, Henan Cancer Hospital, Zhengzhou, China; ^2^ Medical Department, 3D Medicines Inc., Shanghai, China; ^3^ Department of Surgery, The Affiliated Cancer Hospital of Zhengzhou University, Henan Cancer Hospital, Zhengzhou, China

**Keywords:** HER-2, PD-L1, prognosis, gastric cancer, CD8+T cells

In the published article, there was an error regarding the affiliation(s) for Huifang Lv, Keran Sun, Caiyun Nie, Beibei Chen, Jianzheng Wang, Weifeng Xu, Saiqi Wang, and Xiaobing Chen. As well as having affiliation(s) Department of Oncology, Henan Cancer Hospital, Zhengzhou, China, they should also have Department of Oncology, Affiliated Cancer Hospital of Zhengzhou University, Henan Cancer Hospital, Zhengzhou, Henan, China.

In the published article, there was an error regarding the affiliation for Yingjun Liu. As well as having affiliation(s) Department of Surgery, Henan Cancer Hospital, Zhengzhou, China, the author should also have Department of Surgery, Affiliated Cancer Hospital of Zhengzhou University, Henan Cancer Hospital, Zhengzhou, Henan, China.

The authors apologize for these errors and state that these do not change the scientific conclusions of the article in any way. The original article has been updated.

